# S83. THE IMPACT OF COENZYME Q10 ON THE COGNITIVE DEFICITS AND SYMPTOMS OF SCHIZOPHRENIA: PROTOCOL AND BASELINE DATA OF A RANDOMISED, PLACEBO-CONTROLLED STUDY

**DOI:** 10.1093/schbul/sby018.870

**Published:** 2018-04-01

**Authors:** Áine Maguire, April Hargreaves, Michael Gill

**Affiliations:** Trinity College Dublin

## Abstract

**Background:**

CoQ10 is a vital component of mitochondrial function and metabolism, and its deficiency creates greater vulnerability to disease due to impaired mitochondrial energy generation and cellular antioxidant capacity. CoQ10 functions as an electron carrier within the mitochondrial electron transport chain during cellular respiration. Schizophrenia is a disorder with documented CoQ10 deficiency and mitochondrial dysfunction, and cellular respiration and mitochondrial network dynamics can be impaired due to altered complex 1 activity in the disorder. Key features of schizophrenia such as depression, fatigue and cognitive impairment have been independently associated with mitochondrial dysfunction and increased oxidative stress. In bipolar disorder, fibromyalgia, chronic fatigue syndrome and multiple sclerosis these symptoms have been effectively reduced through CoQ10 supplementation. We assess the impact of CoQ10 supplementation in individuals with a diagnosis of schizophrenia through a double-blind, randomised, placebo-controlled study.

**Methods:**

Approximately 300 participants with a DSM-IV diagnosis of schizophrenia or schizoaffective disorder, with no neurological or psychiatric co-morbidity will be recruited to this study. Participants will be randomised to take 100 mg dose capsules of CoQ10 or placebo three times daily for six months, and undergo neuropsychological and cognitive testing at three time points (baseline, midpoint, six months post-randomisation). Changes in participants’ global cognitive function, sustained attention, working memory, processing speed, negative symptoms, levels of depression and anxiety, fatigue, blood pressure, quality of life and functional status following CoQ10 supplementation will be assessed. Blood samples are also taken at each assessment session to assess baseline and changes in levels of plasma CoQ10 and mitochondrial function via lactate analysis.

**Results:**

Currently baseline data is available for 42 participants (mean age = 50.2, SD=10.7). All participants either have a clinical diagnosis of schizophrenia (n=34) or schizoaffective disorder (n=8). The mean estimated IQ of the group is 92.4 (SD=20.5), and participants have a median of 13 years in education. Thirty-nine percent of participants reported mild to severe levels of depression and twenty-three percent reported moderate or severe levels of anxiety. Seventy-three percent of participants reported good to very good quality of life. FACIT-fatigue scores were negatively correlated with both depression and anxiety scores, such that greater fatigue levels were associated with higher levels of depression (r=-.484, p<0.01) and anxiety (r=-.539, p<0.01).

**Discussion:**

CoQ10 is a mitochondrial agent that plays a fundamental role in energy production and mitochondrial function. The available baseline data suggest a relationship between fatigue and depression and anxiety levels in individuals with a diagnosis of schizophrenia. CoQ10 supplementation has the potential to affect these symptoms, through CoQ10’s ability to restore electron flow in the electron transfer chain and increase mitochondrial antioxidant capacity. The study commenced in November 2016 and patient enrolment and assessment is ongoing. Updated baseline information will be presented including further cognitive assessments. To minimise risk of bias while recruitment and assessments are ongoing, unblinding and outcome analysis will not be conducted at time of presentation.

